# Bevacizumab Combined with Corticosteroids Does Not Improve the Clinical Outcome of Nasopharyngeal Carcinoma Patients With Radiation-Induced Brain Necrosis

**DOI:** 10.3389/fonc.2021.746941

**Published:** 2021-09-28

**Authors:** Honghong Li, Xiaoming Rong, Weihan Hu, Yuhua Yang, Ming Lei, Wenjie Wen, Zongwei Yue, Xiaolong Huang, Melvin L. K. Chua, Yi Li, Jinhua Cai, Lei He, Dong Pan, Jinping Cheng, Yaxuan Pi, Ruiqi Xue, Yongteng Xu, Yamei Tang

**Affiliations:** ^1^ Department of Neurology, Sun Yat-sen Memorial Hospital, Sun Yat-sen University, Guangzhou, China; ^2^ Department of Radiation Oncology, Cancer Center of Sun Yat-sen University, Guangzhou, China; ^3^ Department of Intensive Care Unit, The First Affiliated Hospital of Xiamen University, Xiamen, China; ^4^ Oncology Academic Clinical Program, Duke-National University of Singapore (NUS) Medical School, Singapore, Singapore; ^5^ Division of Radiation Oncology, National Cancer Centre Singapore, Singapore, Singapore; ^6^ Division of Medical Sciences, National Cancer Centre Singapore, Singapore, Singapore; ^7^ Guangdong Provincial Key Laboratory of Malignant Tumor Epigenetics and Gene Regulation, Sun Yat-sen Memorial Hospital, Sun Yat-sen University, Guangzhou, China; ^8^ Guangdong Province Key Laboratory of Brain Function and Disease, Zhongshan School of Medicine, Sun Yat-sen University, Guangzhou, China

**Keywords:** radiation-induced brain necrosis, bevacizumab combined with corticosteroid, real-world data, nasopharyngeal carcinoma, bevacizumab monotherapy

## Abstract

**Objective:**

Our aim was to compare the clinical outcomes of patients treated with bevacizumab combined with corticosteroids and those with bevacizumab monotherapy from a radiation-induced brain necrosis (RN) registry cohort (NCT03908502).

**Methods:**

We utilized clinical data from a prospective RN registry cohort (NCT03908502) from July 2017 to June 2020. Patients were considered eligible if they had symptomatic RN after radiotherapy for nasopharyngeal carcinoma (NPC) and received bevacizumab (5 mg/kg, two to four cycles) with a minimum follow-up time of 3 months. The primary outcome was a 2-month response rate determined by MRI and clinical symptoms. Secondary outcomes included quality of life [evaluated by the abbreviated World Health Organization Quality of Life (WHOQOL-BREF) questionnaire] and cognitive function (evaluated by the Montreal Cognitive Assessment scale) at 2 months, RN recurrence during follow-up, and adverse events.

**Results:**

A total of 123 patients (34 in the combined therapy group and 89 in the monotherapy group) were enrolled in our study with a median follow-up time of 0.97 year [interquartile range (IQR) = 0.35–2.60 years]. The clinical efficacy of RN did not differ significantly between patients in these two groups [odds ratio (OR) = 1.642, 95%CI = 0.584–4.614, *p* = 0.347]. Furthermore, bevacizumab combined with corticosteroids did not reduce recurrence compared with bevacizumab monotherapy [hazard ratio (HR) = 1.329, 95%CI = 0.849–2.079, *p* = 0.213]. The most common adverse events of bevacizumab were hypertension (17.89%), followed by nosebleed (8.13%) and fatigue (8.13%). There was no difference in grade 2 or more severe adverse events between the two groups (*p* = 0.811).

**Interpretation:**

Our results showed that the treatment strategy of combining bevacizumab with corticosteroids did not lead to better clinical outcomes for RN patients with a background of radiotherapy for nasopharyngeal carcinoma.

## Introduction

Nasopharyngeal carcinoma (NPC) is characterized by a distinct geographical feature with high incidence rates in southern China and Southeast Asia (1). The age-standardized incidence rate of NPC in China is 3.0 per 100,000 people (2). NPC is highly sensitive to ionizing radiation, and radiotherapy is the mainstay of its treatment strategy. However, patients often suffer from a delayed complication called radiation-induced brain necrosis (RN), occurring 6 months to several years after radiotherapy. The typical symptoms of RN include headache, cognitive dysfunction, dysphagia, and mental disorder, which severely affect patients’ quality of life (3). Efforts to prevent or minimize RN have long been attempted in both laboratory and clinical studies.

However, limited medication strategies are recommended for treating RN patients, and corticosteroids and bevacizumab have been in widespread use (4–6). Corticosteroids were reported with a lower effectiveness rate of 33.3% (7), while that of bevacizumab has reached 65.5% (6). However, bevacizumab was confirmed to have a 25% recurrence rate during the 6-month follow-up compared with 22% in corticosteroids (6). Thus, we wondered whether bevacizumab combined with corticosteroids would increase the efficacy and reduce the recurrence rate. The RN cohort study (NCT03908502) was a prospective observational study on the clinical manifestations, therapeutic effects, and prognosis of radiotherapy-related nervous system complications. The patients were from all over China and mostly from the Sun Yat-sen University Cancer Center, which is the largest center for NPC patients in the world. The annual NPC outpatient number was up to 4,000. Our institution received about 180 radiotherapy-induced brain necrosis patients every year. Therefore, we planned to explore the relationship between the different bevacizumab-associated treatment strategies and prognosis in a large set of real-world data from this registry cohort, which would be greatly helpful for RN treatment.

## Materials and Methods

### Study Design and Patients

For the current study, patients were selected from the RN cohort study according to the following criteria: 1) patients with NPC, treated with radiotherapy, and confirmed RN without evidence of tumor recurrence by using radiographic imaging (magnetic resonance imaging, MRI). The radiographic diagnosis of RN was defined as a high-intensity lesion on T2-weighted imaging (T2WI) and an associated enhancing lesion on T1 post-gadolinium contrast imaging, with characteristic “soap bubble” or “Swiss cheese” enhancement (8). Brain magnetic resonance spectroscopy, PET-CT, or biopsy would be performed in patients with a differential diagnosis of RN/recurrent or progressive brain tumor. 2) Patients who presented with evidence of progressive neurologic signs or symptoms appropriate to the location of RN, such as seizure, hemiparesis, limb numbness, cognitive impairment, and blurred vision, or symptoms that cannot be clearly localized but are clinically attributable to RN, such as headache and dizziness; 3) the interval between radiotherapy and the diagnosis of RN must exceed 6 months in order to exclude acute radiation injury to the brain; 4) patients who received at least two cycles of bevacizumab (5 mg/kg, every 2 weeks); and 5) those with a follow-up duration of more than 3 months.

The RN cohort was a non-interventional study capturing patient and disease characteristics, treatments, and outcomes for RN diagnosed at Sun Yat-sen Memorial Hospital, Sun Yat-sen University. Patients without informed consent were excluded from the cohort. This prospective observational study was approved by the Ethics Committee of our hospital and was registered on clinicaltrials.gov (NCT03908502).

In addition to brain injury, radiotherapy can also cause cranial nerve injury, especially in those who have received re-radiotherapy (9). Radiation-induced cranial nerve injury can occur alone or concurrently with RN. For radiation-induced cranial nerve injury, corticosteroids are recommended. We usually treat cranial nerve injury by using methylprednisolone 80 mg/day (intravenous) for 4 days, followed by 60 mg once daily from days 5 to 7, 40 mg once daily from days 8 to 10, oral prednisone 30 mg/day from days 11 to 17, and gradually tapered by 5 mg/kg and ended by 10 mg daily for 3 months.

The patients included in our study were divided into two groups: 1) bevacizumab monotherapy group and 2) bevacizumab in combination with corticosteroid group. The criteria for treating RN patients with bevacizumab were as follows: no cerebral hemorrhage or bleeding in other organs, negative urinary protein, and no bevacizumab allergy. Patients received 5 mg/kg bevacizumab intravenously. The treatment is repeated every 2 weeks for up to four cycles in the absence of disease progression or unacceptable side effects. For the second group, the combination of corticosteroids was used on the condition of concurrent diagnosis of RN and a newly diagnosed radiation-induced cranial nerve injury. Patients in this group received intravenous bevacizumab at a dose of 5 mg/kg at 2-week intervals for up to four treatments. In addition, patients received corticosteroids at the dose stated above. Brain MRI was performed for all patients at baseline, week 4, and week 8, and when symptoms relapse.

The following demographic and clinical characteristics were recorded: sex, age, clinical symptoms, total radiation dose of the brain and neck, radiotherapy approaches [conventional *vs*. intensity-modulated radiation therapy (IMRT)], duration between radiotherapy and RN diagnosis (DRRN), duration between radiotherapy and bevacizumab treatment (DRT), duration between RN diagnosis and bevacizumab treatment (DRNT), RN treatment strategy, the location of the RN lesion, and the RN volume on MRI at baseline and during follow-up (10, 11). Quality of life assessed by the abbreviated World Health Organization Quality of Life (WHOQOL-BREF) scale (12) and cognitive function assessed by the Montreal Cognitive Assessment (MoCA) scale were also collected at baseline and post-treatment (13).

### Efficacy Assessments

To assess the RN volume, T2 fluid-attenuated inversion recovery (T2-FLAIR) images were analyzed. On T2-FLAIR images, the outline of the RN lesion was first identified using manual and semiautomatic approaches, and then the total volume was automatically calculated with Volume Viewer 2 software (GE Healthcare, AW Suite2.0 6.5.1.z). The primary outcome was an 8-week response rate as determined by MRI and clinical symptoms. We defined clinical effectiveness as a reduction of edema volume on T2WI/FLAIR by ≥25% at week 8 over baseline without deteriorating symptoms. Recurrence was defined as an increase of edema volume on T2WI/FLAIR by ≥10% over that of the last MRI taken after treatment or any new lesion clinically attributed to RN (9).

### Statistical Analysis

Statistical analysis was performed using SPSS 23.0 and R for Windows (version 3.4.2; http://www.r-project.org). Multiple imputations were performed to fill in the missing quality of life (QOL) scores and MoCA scores of 11 patients. Patient characteristics were analyzed using descriptive statistical methods. Data were presented as median (interquartile range) or number (percentage). Categorical variables were compared with chi-square and Fisher’s exact tests. Non-parametric data were compared with the Mann–Whitney *U* test. The efficacy and side effects were analyzed using logistic regression. The recurrence outcome was analyzed using Cox regression analysis. The Kaplan–Meier approach was also chosen to analyze RN recurrence. All analyses were two-sided, and a *p*-value <0.05 was considered to be statistically significant.

## Results

### Clinical Characteristics

**Figure 1:**
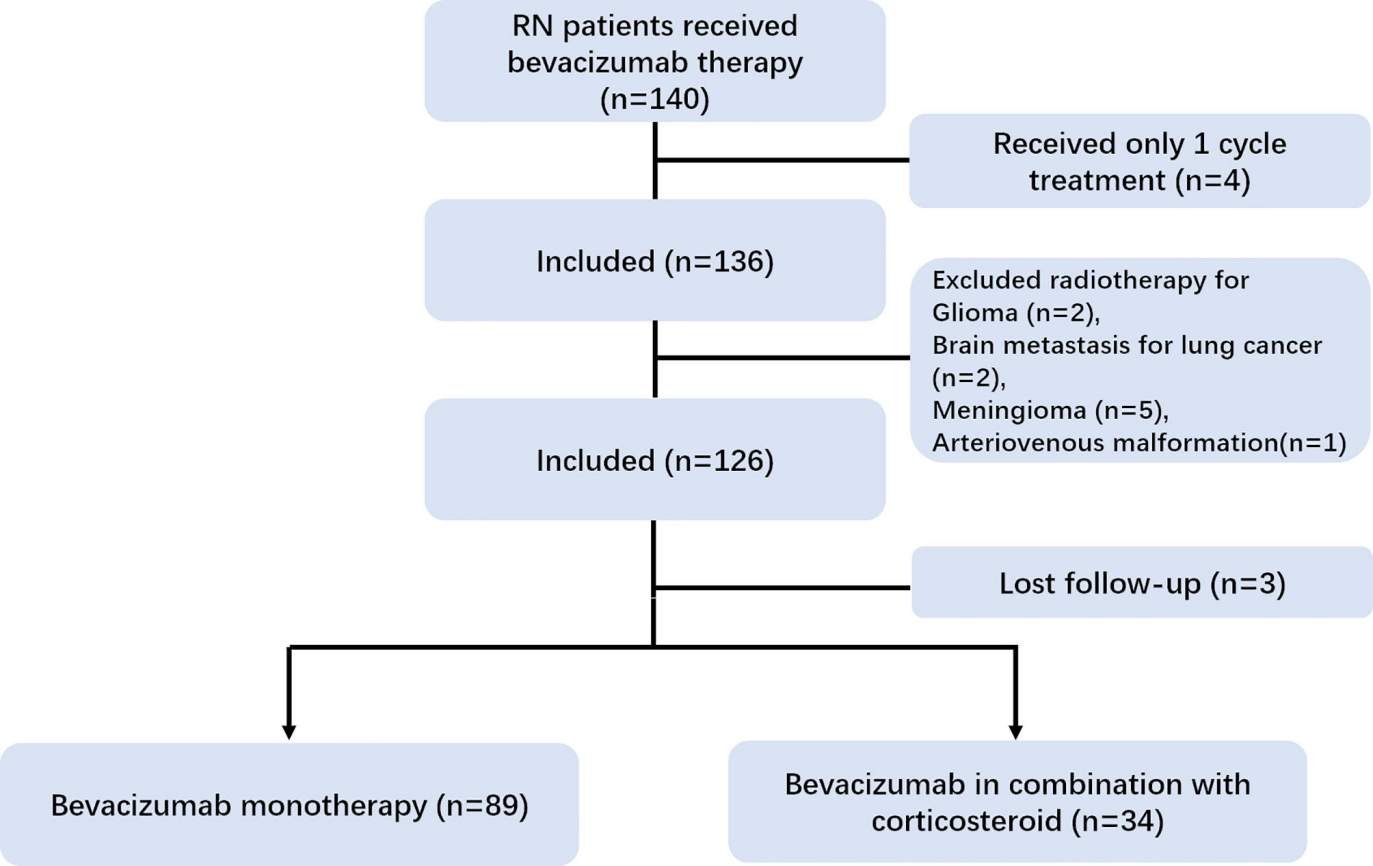
CONSORT diagram.

In our registry cohort, a total of 140 RN patients received bevacizumab therapy. We excluded 10 patients with head and neck cancer other than NPC. Three patients were lost to follow-up and four patients with only one cycle of treatment were further excluded from the analysis. Lastly, 123 patients [median age = 48 years, interquartile range (IQR) = 41–55 years] were enrolled in our study (**Figure 1**). The median DRRN, DRNT, and DRT were 43.89 (IQR = 26.12–61.96 months), 3.58 (IQR = 0.76–11.90 months), and 52.41 months (IQR = 34.03–75.44 months), respectively. The frequency of IMRT was performed in 75 out of 123 (61%) patients.

As for the location of RN lesions, the RN lesions of 37 patients were in the left temporal lobe, 34 in the right temporal lobe, 45 in the bilateral temporal lobe, 4 in the brain stem, and those of 3 patients were in the frontal lobe. Sixty-four (52.0%) out of 123 patients received four cycles of bevacizumab. Forty-eight (39.0%) individuals received only two cycles of bevacizumab as they achieved radiographic evidence of complete remission. Eleven patients discontinued bevacizumab after two cycles of treatment due to adverse events (seven with nosebleed and four with uncontrolled hypertension).

Among the 123 patients, 34 (27.6%) patients who suffered from RN and newly diagnosed cranial nerve injury were treated with bevacizumab combined with corticosteroids; the other 89 (72.4%) patients received bevacizumab monotherapy. The demographic and clinical data are presented in **Table 1**. No significant difference was found in RN volume or radiation-related characteristics at baseline between the two groups (**Table 1**).

**Table 1:** Demographic characteristics.

	All patients (n = 123)	Bevacizumab monotherapy (n = 89)	Combination of bevacizumab with corticosteroid (n = 34)	*P value*
**Demographic and Clinical characteristics**				
**Age**, years	48.00 [41.00, 55.00]	50.00 [42.00, 55.00]	46.00 [39.50, 53.00]	0.133^a^
**Sex**				0.974^b^
Male	92 (74.8)	66 (74.2)	26 (76.5)	
Female	31 (25.2)	23 (25.8)	8 (23.5)	
**Dizziness**	36 (29.3)	24 (27.0)	12 (35.3)	0.493^b^
**Headache**	37 (30.1)	26 (29.2)	11 (32.4)	0.905^b^
**Vision dysfunction**	25 (20.3)	16 (18.0)	9 (26.5)	0.426^b^
**Memory decline**	21 (17.1)	16 (18.0)	5 (14.7)	0.87^c^
**Seizure**	19 (15.4)	14 (15.7)	5 (14.7)	1^c^
**Limb weakness**	5 (4.1)	4 (4.5)	1 (2.9)	1^c^
**Personality change**	7 (5.7)	4 (4.5)	3 (8.8)	0.623^c^
**QOL scores at baseline**	53.88 [47.86, 57.77]	53.88 [48.21, 57.64]	54.37 [47.24, 58.86]	0.682^a^
**MoCA scores at baseline**	25.00 [21.00, 27.00]	25.00 [22.00, 27.00]	23.00 [19.00, 26.00]	0.113^a^
**RN lesion associated characteristics**				
**Lesion**				0.011^c^
Left temporal lobe	37 (30.1)	31 (34.8)	6 (17.6)	
Right temporal lobe	34 (27.6)	17 (19.1)	17 (50.0)	
Bilateral temporal lobe	45 (36.6)	35 (39.3)	10 (29.4)	
Brainstem	4 (3.3)	4 (4.5)	0 (0.0)	
Frontal lobe	3 (2.4)	2 (2.2)	1 (2.9)	
**RN volume at baseline**, cm^3^	33.99 [14.74, 74.31]	35.59 [15.31, 68.51]	31.22 [13.28, 84.18]	0.808^a^
**Radiation-related characteristics**				
**Total radiation dose of the brain**, Gy	70.00 [68.00, 70.00]	70.00 [68.00, 70.00]	70.00 [68.00, 70.00]	0.68^a^
**Total radiation dose of the neck**, Gy	60.00 [59.00, 66.00]	60.00 [56.00, 64.00]	62.00 [60.00, 66.00]	0.111^a^
**Radiotherapy approaches**				0.465^b^
Traditional	48 (39.0)	37 (41.6)	11 (32.4)	
IMRT	75 (61.0)	52 (58.4)	23 (67.6)	
**DRRN**, month	43.89 [27.03, 62.60]	46.95 [26.24, 63.22]	43.32 [29.03, 57.42]	0.767^a^
**DRNT**, month	3.58 [0.76, 11.90]	3.42 [0.66, 11.97]	6.13 [1.05, 10.35]	0.474^a^
**DRT**, month	52.41 [36.08, 77.34]	53.33 [37.61, 78.48]	50.55 [34.11, 74.78]	0.814^a^

RN, radiation-induced brain necrosis; DRRN, duration between radiotherapy and RN diagnosis; DRNT, duration between RN diagnosis and bevacizumab treatment; DRT, duration between radiotherapy and bevacizumab treatment; IMRT, intensity modulated radiation therapy; QOL, WHOQOL-BREF scores; MoCA, Montreal Cognitive Assessment.

^a^Mann-Whitney U test; ^b^Chi-square; ^c^fisher exact test.

### Radiographic and Clinical Efficacy

The percentage decreases in RN volume were 63% (IQR = 29–88) and 61% (IQR = 16–82) in the monotherapy and the combination group, respectively, measured by T2-FLAIR imaging at week 8 over baseline. Bevacizumab combined with corticosteroids did not further reduce the edema volume compared with bevacizumab monotherapy (*p* = 0.615).

The response rate was 76.4% (68/89) in the bevacizumab monotherapy group and was 70.6% (24/34) in the bevacizumab combined with corticosteroid group. Compared with bevacizumab monotherapy, bevacizumab combined with corticosteroids did not further increase the response rate [odds ratio (OR) = 1.642, 95%CI = 0.584–4.614, *p* = 0.347] (**Table 2**).

**Table 2:** Treatment outcome between two groups.

	OR/ HR	95% CI	P value
**Efficacy**			
Bevacizumab monotherapy	Ref		
Combination of bevacizumab with corticosteroid	1.642	[0.584, 4.614]	0.347
**Recurrence**			
Bevacizumab monotherapy	Ref		
Combination of bevacizumab with corticosteroid	1.329	[0.849, 2.079]	0.213
**Adverse events of grade 2 or greater**			
Bevacizumab monotherapy	Ref		
Combination of bevacizumab with corticosteroid	0.706	[0.276, 1.806]	0.468

Multivariate backward stepwise regression analysis based on Akaike Information Criterion (AIC), adjusted for age, sex, DRNR, DTRN, DTR, lesion, total radiation dose of the brain, total radiation dose of the neck, radiotherapy approaches, and RN volume at baseline.

**Figure 2:**
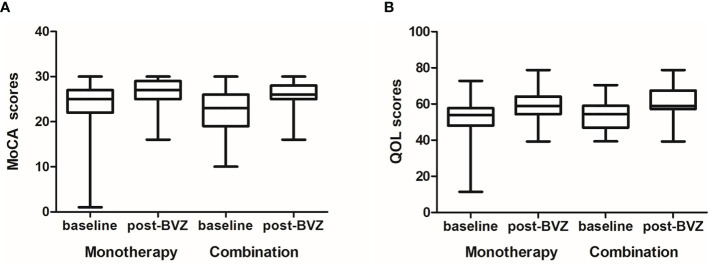
The quality of life (QOL) and Montreal Cognitive Assessment (MoCA) scores at baseline and post-treatment between the two groups. **(A)** The increase of QOL at 2 months over the baseline was similar in the two groups (5.12 *vs*. 6.69, *p* = 0.674). **(B)** As for cognitive function assessment, no significant difference in the change of MoCA scores was found between the monotherapy and the combination group (2.0 *vs*. 3.0, *p* = 0.138).

Furthermore, score improvements on the QOL scale were achieved in 87.6% (78/89) and 88.2% (30/34) of patients in the monotherapy and the combination group, respectively, at 2 months. The increase of QOL at 2 months over baseline was similar in the two groups (5.12 *vs*. 6.69, *p* = 0.674). As for cognitive function assessment, patients on bevacizumab monotherapy had a median improved MoCA score of 2.0 following 2 months of treatment. No significant difference in the change of MoCA score was found between the monotherapy and combination groups (2.0 *vs*. 3.0, *p* = 0.138) (**Figure 2**).

### Necrosis Recurrence

**Figure 3:**
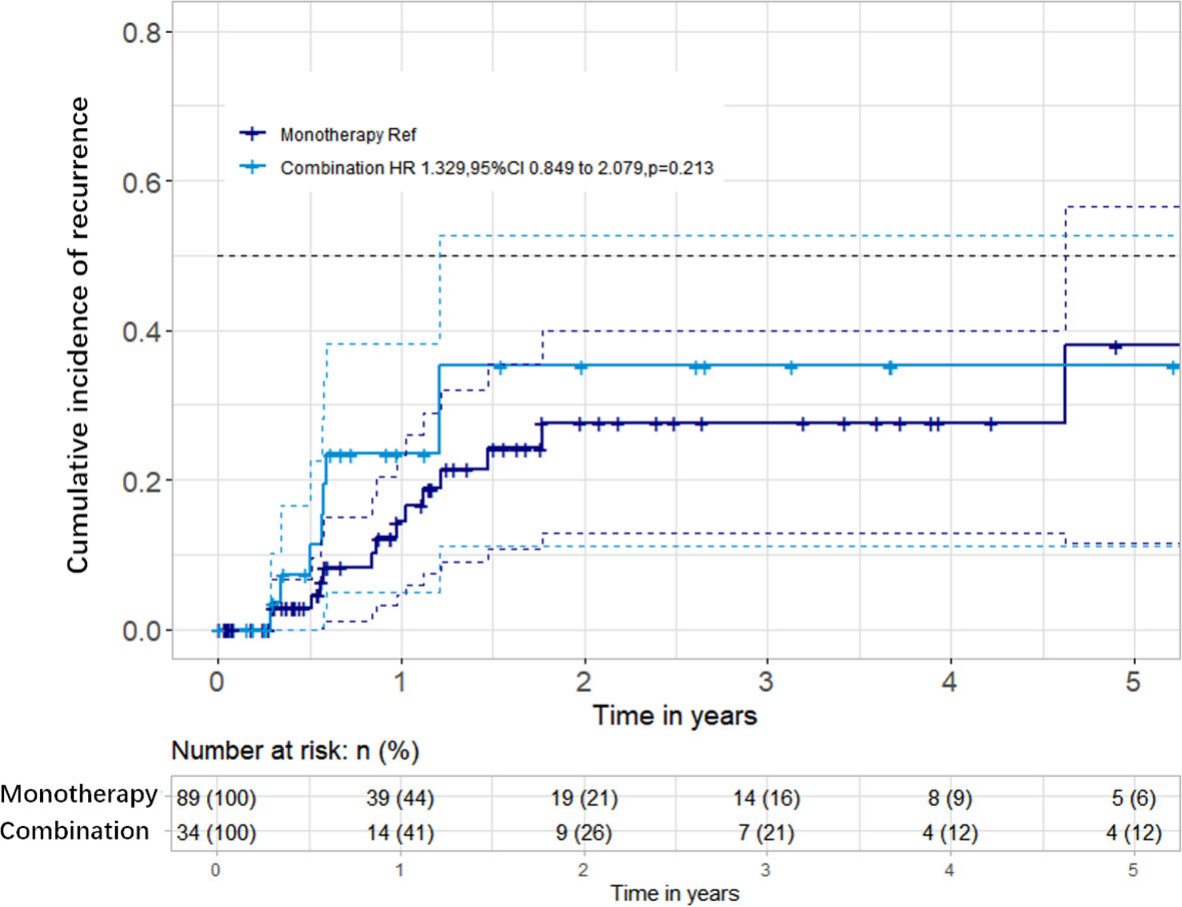
Cumulative incidence of radiation-induced brain necrosis (RN) recurrence between the two groups. There were no significant differences in the recurrence rates between the bevacizumab monotherapy and bevacizumab combined with corticosteroid groups.

The median follow-up time was 0.97 year (IQR = 0.35–2.60 years). There was no significant difference in the follow-up time between the two groups (0.97 *vs*. 0.95, *p* = 0.257). During follow-up, 23 patients (14 in the bevacizumab monotherapy group and 9 in the bevacizumab combined with corticosteroid group) presented new or deteriorating neurologic symptoms and brain MRI recurrence. The median recurrent times were 0.87 and 0.70 year in the monotherapy and combination groups, respectively. Kaplan–Meier survival analysis was conducted to establish the cumulative incidence of recurrence; no difference was found in the two groups (*p* = 0.213) (**Figure 3**). Cox regression analyses, after adjusting for confounding factors, showed that bevacizumab combined with corticosteroid did not reduce recurrence compared with bevacizumab monotherapy [hazard ratio (HR) = 1.329, 95%CI = 0.849–2.079, *p* = 0.213] (**Table 2**).

### Treatment-Related Adverse Events

Among the 123 patients in our study, 74 patients suffered from treatment-related adverse reactions, only one of whom had a grade 4 adverse event of intracranial hemorrhage and five had grade 3 adverse events (**Table 3**). The most common adverse events of bevacizumab were hypertension (17.89%), followed by nosebleed (8.13%) and fatigue (8.13%). Grade 2 or greater adverse events did not differ significantly among patients on different bevacizumab treatment strategies (OR = 0.706, 95%CI = 0.276–1.806, *p* = 0.0.468) (**Table 2**).

**Table 3:** Treatment-related adverse events between two groups.

Adverse events	Bevacizumab monotherapy (n = 89)		Combination of bevacizumab with corticosteroid (n = 34)	
	Grade 1	Grade 2 or greater	Grade 1	Grade 2 or greater
Hypertension, *n (%)*	6 (6.74)	11 (12.36)	1 (2.94)	4 (11.76)
Fatigued, *n (%)*	8 (8.99)		2 (5.88)	
Infection, *n (%)*	1 (1.12)	4 (4.49)	1 (2.94)	1 (2.94)
Nosebleed, *n (%)*	3 (1.12)	2 (1.12)	3 (8.82)	2 (5.88)
Intracranial hemorrhage, *n (%)*		1 (1.12)		
Insomnia, *n (%)*	1 (1.12)	4 (4.71)		
Headache, *n (%)*	2 (1.12)			2 (5.88)
Rash, *n (%)*		2 (1.12)	1 (2.94)	1 (2.94)
Fever, *n (%)*				1 (2.94)
Blurred vision, *n (%)*	1 (1.12)			
Hyperglycemia, *n (%)*	1 (1.12)	1 (1.12)		
Ischemic stroke, *n (%)*		1 (1.12)		
Hoarse, *n (%)*		2 (1.12)	3 (8.82)	
Facial paralysis, *n (%)*		1 (1.12)	1 (2.94)	
Swallowing dysfunction, *n (%)*		3 (1.12)	3 (8.82)	
Weight gain, *n (%)*				1 (2.94)
Gastrohelcosis, *n (%)*				1 (2.94)

## Discussion

The treatment of RN is a challenging issue as the number of RN patients has gone up with the widespread application of radiotherapy over the last decades. Corticosteroids and bevacizumab are the two main recommended treatments (13, 14). Corticosteroids are the first-line treatment for RN. A recent review summarized that bevacizumab was more efficacious than corticosteroid treatment (13). Interestingly, our study found that the treatment strategy combining bevacizumab with corticosteroids did not lead to better clinical outcomes for NPC patients with RN.

The mechanism of RN is multifactorial, involving excessive inflammatory response, oxidative stress, microvascular injury, neurogenesis reduction, demyelination, and the decline of glia cells (15). Bevacizumab is a recombinant human monoclonal antibody targeting vascular endothelial growth factor (VEGF) receptors on the surface of endothelial cells, which could restore vascular permeability and reduce brain edema (14). On the other hand, corticosteroids could relieve excessive inflammation and reduce vasogenic edema (16). Besides, corticosteroids could also participate in the repair of the microvasculature endothelial junctions. The possible reasons why the combination of bevacizumab and corticosteroids did not improve effectiveness in our cohort could be the following: firstly, bevacizumab and corticosteroids might share common pathways in treating RN, but do not have additive effects; secondly, a modest increase in effectiveness might have been ignored in our study due to the relatively small sample size. Our results are actually in line with the analysis done by Ahluwalia et al. based on a similar patient cohort (17). Twenty-four patients were enrolled in his study, which demonstrated that bevacizumab resulted in excellent clinical and radiological responses. Twenty-one patients receiving dexamethasone treatment had dose reductions after the initiation of bevacizumab. This study might suggest that bevacizumab did not require combined steroid therapy for RN. Another study showed that the combination of intravitreal anti-VEGF and triamcinolone acetonide treatment did not yield any significant benefits in chronic diabetic macular edema compared to anti-VEGF monotherapy (18). These studies suggested that bevacizumab combined with steroids might not have an enhanced efficacy in radiation-associated injury.

Additionally, we observed that in 39.0% (48/123) of patients with short-term application of bevacizumab, their cerebral lesions almost disappeared after two cycles of treatment. A similar result was seen in a smaller sample size of 21 RN patients (19). In this study, the bevacizumab dosage was 7.5 mg/kg every 3 weeks, and the median number of treatment cycles was 2 (range = 1–4). This study suggested that 52.4% (11/21) of patients who received two cycles showed sufficient improvement. These results suggested that bevacizumab could have a rapid onset of therapeutic effect, while its accumulative effect after two cycles seemed limited. Taken together, an evaluation should be considered in cycle 2 in order to avoid unnecessary medication. Dashti et al. reported two pediatric RN patients who gained greater than 70% reduction of brain edema following only a single administration of 2.5 mg/kg bevacizumab after stereotactic radiosurgery for cerebral arteriovenous malformations (20). The optimal treatment regimen for RN still needs to be explored.

To our knowledge, our study on 123 patients treated with bevacizumab is the largest analysis performed in the RN cohort so far. We used real-world data, which improves the generalizability of the findings and transcends the limited settings in a clinical trial.

There are several limitations in our study. Firstly, the study was done at a single center, so further studies are needed to involve more institutions in order to eliminate potential bias. Secondly, we only enrolled patients who received bevacizumab at a dose of 5 mg/kg every 2 weeks in our study. Varied dosages of bevacizumab, such as 2.5 and 7.5 mg/kg, may produce different outcomes. Further studies regarding different doses and cycles are warranted to discover the optimal bevacizumab strategy for RN.

## Conclusion

The current study demonstrated that the treatment strategy of combining bevacizumab with corticosteroids did not lead to better clinical outcomes for NPC patients with RN. The optimal treatment regimen for RN still needs to be explored.

## Data Availability Statement

Publicly available datasets were analyzed in this study. The datasets used and analyzed during the current study are available from the corresponding author on reasonable request.

## Ethics Statement

The studies involving human participants were reviewed and approved by the Institutional Research Ethics Committee of Sun Yat-sen Memorial Hospital. The patients/participants provided written informed consent to participate in this study.

## Author Contributions

HL was a major contributor in writing the manuscript and acquisition of data. XR and WH analyzed and interpreted the data. ZY, XH, MC, YL, JHC, LH, YX, DP, JPC, YP, and RX performed the data collection and revised the manuscript for important intellectual content. YY, ML, WW revised and edited the English language. YT designed the study, revised the manuscript, and gave final approval of the version to be submitted. All authors contributed to the article and approved the submitted version.

## Funding

This study was funded by the National Science Fund for Distinguished Young Scholars (81925031), Projects of International Cooperation and Exchanges NSFC (81820108026), and the Science and Technology Program of Guangzhou (202007030001) to YT in the design of the study and the collection, analysis, and interpretation of data; the National Natural Science Foundation of China (82003389) to HL; Young Teacher Training Program of Sun Yat-sen University (20ykpy106) to XR; and the National Natural Science Foundation of China (81872549), Guangdong–Hong Kong–Macao Greater Bay Area Center for Brain Science and Brain-Inspired Intelligence Fund (2019012) to YL in writing the manuscript. MC is supported by the National Medical Research Council Singapore Clinician Scientist Award (NMRC/CSA-INV/0027/2018), National Research Foundation Proton Competitive Research Program (NRF-CRP17-2017-05), Ministry of Education Tier 3 Academic Research Fund (MOE2016-T3-1-004), the Duke-NUS Oncology Academic Program Goh Foundation Proton Research Programme, NCCS Cancer Fund, and the Kua Hong Pak Head and Neck Cancer Research Programme.

## Conflict of Interest

MC reports personal fees from Astellas, Janssen, Bayer, Pfizer, MSD, personal fees and non-financial support from AstraZeneca, personal fees and grants from Ferring, personal fees and non-financial support from Varian, non-financial support from Decipher Biosciences, non-financial support from MedLever, and consults for immunoSCAPE Inc., outside the submitted work.

The remaining authors declare that the research was conducted in the absence of any commercial or financial relationships that could be construed as a potential conflict of interest.

## Publisher’s Note

All claims expressed in this article are solely those of the authors and do not necessarily represent those of their affiliated organizations, or those of the publisher, the editors and the reviewers. Any product that may be evaluated in this article, or claim that may be made by its manufacturer, is not guaranteed or endorsed by the publisher.

## Abbreviations

RN, radiation-induced brain necrosis; NPC, nasopharyngeal carcinoma; MRI, magnetic resonance imaging; IMRT, intensity-modulated radiation therapy; DRT, duration between radiotherapy and bevacizumab treatment; DRNT, duration between RN diagnosis and bevacizumab treatment; DRRN, duration between radiotherapy and RN diagnosis; VEGF, vascular endothelial growth factor; QOL, WHOQOL-BREF scores; MoCA, Montreal Cognitive Assessment.

## References

[1] Chen YP Chan A Le QT Blanchard P Sun Y Ma J. Nasopharyngeal Carcinoma. Lancet (2019) 394(10192):64–80. doi: 10.1016/S0140-6736(19)30956-0 31178151

[2] Bray F Ferlay J Soerjomataram I Siegel RL Torre LA Jemal A. Global Cancer Statistics 2018: GLOBOCAN Estimates of Incidence and Mortality Worldwide for 36 Cancers in 185 Countries. CA Cancer J Clin (2018) 68(6):394–424. doi: 10.3322/caac.21492 30207593

[3] Cheung MC Chan AS Law SC Chan JH Tse VK. Impact of Radionecrosis on Cognitive Dysfunction in Patients After Radiotherapy for Nasopharyngeal Carcinoma. Cancer-Am Cancer Soc (2003) 97(8):2019–26. doi: 10.1002/cncr.11295 12673733

[4] Levin VA Bidaut L Hou P Kumar AJ Wefel JS Bekele BN. Randomized Double-Blind Placebo-Controlled Trial of Bevacizumab Therapy for Radiation Necrosis of the Central Nervous System. Int J Radiat Oncol Biol Phys (2011) 79(5):1487–95. doi: 10.1016/j.ijrobp.2009.12.061 PMC2908725 20399573

[5] Boothe D Young R Yamada Y Prager A Chan T Beal K. Bevacizumab as a Treatment for Radiation Necrosis of Brain Metastases Post Stereotactic Radiosurgery. Neuro Oncol (2013) 15(9):1257–63. doi: 10.1093/neuonc/not085 PMC3748921 23814264

[6] Xu Y Rong X Hu W Huang X Li Y Zheng D. Bevacizumab Monotherapy Reduces Radiation-Induced Brain Necrosis in Nasopharyngeal Carcinoma Patients: A Randomized Controlled Trial. Int J Radiat Oncol Biol Phys (2018) 101(5):1087–95. doi: 10.1016/j.ijrobp.2018.04.068 29885994

[7] Lee AW Ng SH Ho JH Tse VK Poon YF Tse CC. Clinical Diagnosis of Late Temporal Lobe Necrosis Following Radiation Therapy for Nasopharyngeal Carcinoma. Cancer-Am Cancer Soc (1988) 61(8):1535–42. doi: 10.1002/1097-0142(19880415)61:8<1535::AID-CNCR2820610809>3.0.CO;2-E 3349419

[8] Greene-Schloesser D Robbins ME Peiffer AM Shaw EG Wheeler KT Chan MD. Radiation-Induced Brain Injury: A Review. Front Oncol (2012) 2:73. doi: 10.3389/fonc.2012.00073 22833841 PMC3400082

[9] Rong X Tang Y Chen M Lu K Peng Y. Radiation-Induced Cranial Neuropathy in Patients With Nasopharyngeal Carcinoma. A Follow-Up Study. Strahlenther Onkol (2012) 188(3):282–6. doi: 10.1007/s00066-011-0047-2 22314578

[10] Cai J Zheng J Shen J Yuan Z Xie M Gao M. A Radiomics Model for Predicting the Response to Bevacizumab in Brain Necrosis After Radiotherapy. Clin Cancer Res (2020) 26(20):5438–47. doi: 10.1158/1078-0432.CCR-20-1264 32727886

[11] Li Y Huang X Jiang J Hu W Hu J Cai J. Clinical Variables for Prediction of the Therapeutic Effects of Bevacizumab Monotherapy in Nasopharyngeal Carcinoma Patients With Radiation-Induced Brain Necrosis. Int J Radiat Oncol Biol Phys (2018) 100(3):621–9. doi: 10.1016/j.ijrobp.2017.11.023 29413276

[12] Li H Li L Huang X Li Y Zou T Zhuo X. Radiotherapy-Induced Dysphagia and its Impact on Quality of Life in Patients With Nasopharyngeal Carcinoma. Strahlenther Onkol (2019) 195(6):457–67. doi: 10.1007/s00066-018-01421-6 30689027

[13] Liao G Khan M Zhao Z Arooj S Yan M Li X. Bevacizumab Treatment of Radiation-Induced Brain Necrosis: A Systematic Review. Front Oncol (2021) 11:593449. doi: 10.3389/fonc.2021.593449 33842309 PMC8027305

[14] Zhuang H Shi S Yuan Z Chang JY. Bevacizumab Treatment for Radiation Brain Necrosis: Mechanism, Efficacy and Issues. Mol Cancer (2019) 18(1):21. doi: 10.1186/s12943-019-0950-1 30732625 PMC6367784

[15] Balentova S Adamkov M. Molecular, Cellular and Functional Effects of Radiation-Induced Brain Injury: A Review. Int J Mol Sci (2015) 16(11):27796–815. doi: 10.3390/ijms161126068 PMC4661926 26610477

[16] Tada E Matsumoto K Kinoshita K Furuta T Ohmoto T. The Protective Effect of Dexamethasone Against Radiation Damage Induced by Interstitial Irradiation in Normal Monkey Brain. Neurosurgery (1997) 41(1):209–17, 217-19. doi: 10.1097/00006123-199707000-00033 9218309

[17] Sadraei NH Dahiya S Chao ST Murphy ES Osei-Boateng K Xie H. Treatment of Cerebral Radiation Necrosis With Bevacizumab: The Cleveland Clinic Experience. Am J Clin Oncol (2015) 38(3):304–10. doi: 10.1097/COC.0b013e31829c3139 23799286

[18] Ahmadieh H Ramezani A Shoeibi N Bijanzadeh B Tabatabaei A Azarmina M. Intravitreal Bevacizumab With or Without Triamcinolone for Refractory Diabetic Macular Edema; a Placebo-Controlled, Randomized Clinical Trial. Graefes Arch Clin Exp Ophthalmol (2008) 246(4):483–9. doi: 10.1007/s00417-007-0688-0 17917738

[19] Bodensohn R Hadi I Fleischmann DF Corradini S Thon N Rauch J. Bevacizumab as a Treatment Option for Radiation Necrosis After Cranial Radiation Therapy: A Retrospective Monocentric Analysis. Strahlenther Onkol (2020) 196(1):70–6. doi: 10.1007/s00066-019-01521-x 31586230

[20] Dashti SR Spalding A Kadner RJ Yao T Kumar A Sun DA. Targeted Intraarterial Anti-VEGF Therapy for Medically Refractory Radiation Necrosis in the Brain. J Neurosurg Pediatr (2015) 15(1):20–5. doi: 10.3171/2014.9.PEDS14198 25360851

